# Changes in diet quality in a randomized weight loss trial in breast cancer survivors: the lifestyle, exercise, and nutrition (LEAN) study

**DOI:** 10.1038/npjbcancer.2016.26

**Published:** 2016-08-24

**Authors:** Chelsea Anderson, Maura Harrigan, Stephanie M George, Leah M Ferrucci, Tara Sanft, Melinda L Irwin, Brenda Cartmel

**Affiliations:** 1 Department of Chronic Disease Epidemiology, Yale School of Public Health, New Haven, CT, USA; 2 Office of Disease Prevention, National Institutes of Health, Bethesda, MD, USA; 3 Department of Internal Medicine, Yale Cancer Center, New Haven, CT, USA; 4 Yale School of Medicine, New Haven, CT, USA

## Abstract

Obesity is associated with increased breast cancer recurrence and mortality. Though some post-diagnosis weight loss interventions have achieved weight loss outcomes, it is unclear whether they also improve diet quality. In the Lifestyle, Exercise, and Nutrition (LEAN) study, overweight or obese breast cancer survivors were randomized to either usual care group (*n*=33) or the 6-month lifestyle intervention (*n*=67). Dietary intake was assessed at baseline and 6 months using a validated food frequency questionnaire, and overall diet quality was calculated using the Healthy Eating Index (HEI)-2010 (range 0–100). Intervention effects on diet were evaluated with generalized linear models. Among the 81 participants (51 intervention, 30 usual care) with dietary data, the mean baseline HEI score was 70.5 (s.d.=8.8) and was improved at 6 months (intervention group=6.8 point increase vs usual care=3.1, *P*=0.09). Intervention group participants achieved greater reductions in percent of energy from total fat (−4.2% vs −1.2%; *P*=0.013) and saturated fat (−2.2% vs −1.1%; *P*=0.003), and greater increases in fiber (4.8 g per 1000 kcal vs 1.3 g per 1000 kcal; *P*=0.007) and fruit (0.5 servings vs 0.0 servings; *P*=0.006) intake. Intervention group participants who lost ⩾5% body weight (*n*=27) demonstrated significantly greater improvements in HEI score (10.4 vs 2.8) than those who lost <5% (*n*=23). The intervention increased fruit and fiber intake and decreased percent energy from fat, and those with greater weight loss achieved greater increases in overall diet quality. These findings support the ability of a weight loss intervention to improve diet among breast cancer survivors.

## Introduction

With advances in early detection and treatment, the 5-year survival rate for breast cancer has increased to almost 90% for all stages combined.^1^ Yet despite these gains, breast cancer remains the second leading cause of cancer deaths among US women.^2^ Among breast cancer survivors, obesity at diagnosis is associated with an increased risk of disease recurrence and both all-cause and breast cancer-specific mortality.^3^ Weight gain post diagnosis may also be associated with a poor prognosis.^4–6^ The majority of breast cancer patients gain weight while undergoing treatment, and many continue to gain weight in the years following diagnosis.^7^ Breast cancer survivors are also at an increased risk of cardiovascular disease and diabetes relative to the general population, both conditions related to being overweight or obese.^8^ Weight loss and/or weight maintenance is thus an important goal for women following breast cancer diagnosis.

A recent systematic review documented the feasibility and effectiveness of comprehensive weight loss interventions for breast cancer survivors.^9^ Although successful interventions have included a dietary component, it is unclear whether weight loss is accompanied by improvements in diet quality or simply a decrease in caloric intake and increase in energy expenditure. A focus on diet quality, as opposed to individual foods and nutrients, accounts for the potentially synergistic effects of foods and food patterns on health.^10^ The Healthy Eating Index 2010 (HEI-2010) aligns with the 2010 Dietary Guidelines for Americans and has been found to be a valid and reliable measure of diet quality.^11^ Though research to date is limited, existing studies suggest that higher diet quality is not associated with breast cancer mortality, but is associated with a reduction in deaths from non-breast cancer causes among breast cancer survivors.^12–14^ While some previous studies have assessed post-intervention changes in dietary components among breast cancer survivors,^15–22^ few have evaluated changes in overall diet quality.^23,24^ This is an important area to explore, as changes in diet quality may be independently associated with improvements in the long-term health of breast cancer survivors.

The Lifestyle, Exercise, and Nutrition (LEAN) study was a randomized controlled trial of a 6-month weight loss intervention among overweight and obese breast cancer survivors.^25^ Modeled after the successful Diabetes Prevention Program (DPP),^26^ the LEAN intervention consisted of a series of either in-person or telephone-based counseling sessions that encouraged weight loss through both physical activity and achieving or maintaining a healthy diet. For the primary outcome, participants randomized to the LEAN intervention achieved significantly greater weight loss than those assigned to a control group (6.4% vs 2.0%), as has been reported previously.^25^ In this report, we present change in diet quality and specific dietary components over the 6-month LEAN study in the intervention group compared with the control group.

## Results

Baseline characteristics by intervention group are presented in Table 1. In both the usual care and intervention groups, the majority of participants were white, married, postmenopausal, and well-educated. Baseline characteristics were similar between the intervention and the usual care groups with the exception of alcohol intake, which was significantly higher in the intervention group (*P*<0.008). Over the 6-month intervention, average percentage of body weight lost was 6.4% in the intervention group and 2.0% in the usual care group (*P*<0.01).

Table 2 shows the average HEI score and individual dietary components by group at baseline and 6 months. At baseline, the average HEI score was 70.17 (s.d.=8.51) in the intervention group and 71.08 (s.d.=9.27) in the usual care group (*P*=0.788). At 6 months, the HEI score improved by 6.80 (s.d.=10.06) in the intervention group. This was nearly double the 3.05 (s.d.=8.03) increase in the usual care group, though the difference in change in HEI score between groups was not statistically significant (*P*=0.09).

Significant differences between groups were observed for change over the 6-month study for four of the eight diet components targeted by our intervention (Table 2). Relative to the usual care group, the intervention group reported greater reductions in percent energy from total fat and from saturated fat. Specifically, percent energy from total fat decreased by 4.19% (s.d.=6.49) in the intervention group, as compared with a 1.12% (s.d.=6.72) reduction in the usual care group (*P*=0.01). Percent energy from saturated fat decreased by 2.20% (s.d.=2.39) in the intervention group and by 1.05% (s.d.=2.16) in the usual care group (*P*=0.003). The intervention group also reported greater increases in fiber intake and fruit servings at 6 months. Fiber grams increased by 4.84 g per 1,000 kcal (s.d.=4.72) among the intervention group and 1.28 g per 1,000 kcal (s.d.=4.21) among the usual care group (*P*=0.007). Fruit servings increased by 0.66 servings (s.d.=1.66) in the intervention group and decreased by 0.37 servings (s.d.=1.51) in the usual care group (*P*=0.006). For the remaining dietary intake variables—including calories, fat grams, added sugar, and vegetables servings—the intervention group improved to a greater extent than the usual care group, but differences between groups were not statistically significant.

Within group associations between percent weight loss and change in HEI score are shown in Table 3. There was a significant correlation between percent weight loss and change in HEI in the intervention group (*r*=−0.42 *P*=0.002) with the increase in HEI score greater among participants who lost at least 5% of their initial body weight, relative to those who lost <5% (10.35 vs 2.82, *P*=0.01).

## Discussion

This study evaluated the effect of a 6-month lifestyle and weight loss intervention on diet quality and selected dietary components among overweight and obese breast cancer survivors. Results demonstrate that the LEAN intervention, which resulted in an average 6.4% weight loss,^25^ was successful in increasing fruit and fiber consumption and in decreasing percent energy from both fat and saturated fat. Though differences between intervention and usual care groups were not significant, changes in diet quality as assessed by the HEI-2010 were in the expected direction. Of note, intervention participants who lost more weight had greater increases in HEI score, suggesting that improvements in overall diet quality were an additional benefit of the intervention among women who achieved successful weight loss.

Previous reports have documented post-intervention changes in other diet quality indices among survivors of breast cancer and other cancer types.^23,24,27–29^ However, this report is unique for the focus on change in the HEI-2010, the most recent version of the HEI, in addition to the change in specific dietary components frequently assessed in weight loss intervention studies. The HEI emphasizes consumption of more fruits, vegetables, and whole grains and less refined grains, sodium, and empty calories.^30^ High scores on the HEI reflect diets with an appropriate balance of food groups, rather than individual nutrients, thus allowing for the potentially synergistic effects of individual dietary components to be evaluated. The average HEI score among LEAN participants at baseline was ~70, comparable to that found among participants who voluntarily enroll in large diet studies, such as the NIH-AARP Diet and Health Study.^31^ On the other hand, a recent study using data from the National Health and Nutrition Examination Survey (NHANES) reported an average HEI-2010 score of only 51 among breast cancer survivors,^32^ suggesting that LEAN participants were already eating relatively healthy diets.

In the general population, higher scores on the HEI-2010 have been associated with reduced all-cause mortality, as well as cardiovascular disease and cancer-specific mortality among women.^31,33,34^ Using the previous version of the HEI, the HEI-2005, an inverse association between post-diagnosis diet quality and all-cause mortality has also been documented among breast cancer survivors.^14^ However, the relationship between diet quality and breast cancer-specific survival remains unclear, as few studies have examined such associations.^12–14^ To our knowledge, the impact of post-diagnosis changes in diet quality on breast cancer outcomes has also not been explored. At 6 months, participants in the LEAN intervention improved their HEI-2010 score by an average of 6.8 points, and those who lost at least 5% of their initial body weight demonstrated an increase of over 10 points. Further studies are needed to evaluate the impact of such changes—both alone and in conjunction with weight loss—on outcomes among breast cancer survivors.

Notably, we also observed significant changes in components of the diet that were specifically emphasized in the structure of the LEAN intervention. Decreasing energy intake from fat was emphasized as a way to reduce caloric intake and promote weight loss, while the increases in fruit and fiber consumption were consistent with the recommendation to eat a predominately plant-based diet. Though not significantly different from the usual care group, other aspects of diet, including intakes of vegetables and added sugars, also improved in the intervention group compared with the usual care group. While some studies have suggested that diets low in saturated fat and high in fruits and vegetables are associated with improved breast cancer prognosis and survival, such associations have not been demonstrated consistently.^35,36^ However, such diets have been shown to decrease mortality from heart disease,^31,33,34^ the most common cause of death in breast cancer survivors.^37^

Though this study has several strengths, including the randomized design and counseling from a registered dietician to target dietary changes, there are also potential limitations. One limitation is measurement error inherent to the food frequency questionnaire (FFQ).^38^ In addition, the use of an FFQ to assess dietary change may lead to differential reporting between intervention and control groups. LEAN participants were also fairly homogenous with respect to sociodemographic characteristics such as age and race/ethnicity, and thus our results may not be generalizable to other more diverse groups of breast cancer survivors. Furthermore, the LEAN study was not powered to detect changes in diet quality, as this was not a primary outcome. Larger studies are needed to evaluate the impact of weight loss interventions, such as the LEAN intervention, on HEI scores and ultimately the impact of diet quality on recurrence and survival.

Even though LEAN participants may have been eating a healthier diet compared with the general population, as evidenced by their relatively high HEI scores at enrollment, we still saw modest improvements in the intervention group for HEI score and significant improvements for diet components targeted by our intervention. These dietary changes could be attributed to the fact that women who volunteer for intervention trials are also often more educated and more motivated than a more general population sample. However, a breast cancer diagnosis may represent a teachable moment, and thus many survivors may be highly motivated to make lifestyle changes, such as improving their diet.

In summary, the efficacious LEAN weight loss intervention improved several aspects of diet among breast cancer survivors. The changes observed reflect key recommendations of the dietary counseling in the intervention. Diet quality also changed favorably among intervention participants, particularly those who lost a greater percentage of their body weight. These findings suggest that weight loss interventions can favorably impact participants’ diet quality, with potential implications for the overall health and longevity of breast cancer survivors. Future trials should be designed to assess the impact of dietary change on long-term outcomes such as recurrence and survival.

## Materials and methods

### Participants

Women who had a body mass index (BMI)⩾25.0 kg/m^2^ and had been diagnosed with Stage 0 to III breast cancer in the 5 years prior to enrollment were eligible for the study. Eligible participants also had completed chemotherapy and/or radiation and were physically able to exercise. Women had to be accessible by telephone and be able to read and communicate in English. Details of recruitment procedures can be found elsewhere.^25^ Briefly, the study was advertised in the Breast Center at Smilow Cancer Hospital at Yale-New Haven and the Yale Cancer Center Survivorship Clinic. Women self-referred and were recruited between 1 June 2011 and 30 December 2012. The study was approved by the Yale School of Medicine Human Investigation Committee.

One hundred eligible women agreed to randomization and gave informed consent. Using a random permuted block design, participants were randomly assigned to one of the following groups: in-person counseling, telephone-based counseling, or usual care. Eighty-one women (81%), (51 intervention, 30 usual care) had complete dietary data and were included in the analysis.

### Intervention groups

For both the in-person and telephone-based counseling groups, the intervention consisted of 11 individualized sessions with a registered dietician. The content of the weight loss program was similar for both groups, with only the delivery being different (in-person vs telephone counseling). The sessions, ~30 min in duration, occurred each week for the first month, followed by bimonthly sessions in months 2 and 3 and monthly sessions in months 4–6.

The intervention components were adapted from the DPP,^26^ as well as weight management materials from the American Institute for Cancer Research (AICR), the American Cancer Society, and the National Cancer Institute. Dietary recommendations were also drawn from the Dietary Guidelines 2010.^39^ The primary goal of the intervention was for participants to achieve a weight loss of 10% of initial body weight. A combination of reduced caloric intake, increased physical activity, and behavior therapy was emphasized, with strategies based on constructs of the Social Cognitive Theory. For the diet portion of the intervention, participants set goals for energy intake in the range of 1,200–2,000 kcal per day (based on baseline weight) and were advised to limit fat consumption to 25% of total energy intake. Other targets included increasing fiber intake to 25 g per day and reducing consumption of added sugars. Eating a largely plant-based diet was also emphasized, with the recommendations to eat a minimum of five servings of fruits and vegetables per day, and to reduce portion sizes of meat and other animal products.

To track their personal intake of various dietary components, such as fat and fiber, women in the intervention groups recorded their daily food consumption in a log book developed for the study. The women were also asked to weigh themselves weekly at home using a scale provided for them.

### Usual care group

Participants assigned to the usual care group received AICR nutrition and physical activity brochures. They were also referred to the Yale Cancer Center Survivorship Clinic, which offers a two-session weight management program. Following study completion at 6 months, participants in the usual care group were offered all educational material provided to participants in the counseling groups, as well as an in-person counseling session.

### Assessments

Medical history and demographic data were collected via questionnaire at baseline. Anthropometric measures, including weight and height, were assessed by study staff during clinic visits at both baseline and 6 months.

Dietary intake was assessed at baseline and 6 months using a 120-item FFQ. This instrument was developed for the Women’s Health Initiative (WHI) and has been validated against 4-day food records and 24-h dietary recalls.^40^ The nutrient database used to analyze the WHI FFQ was derived from the Nutrition Data Systems for Research, version 2005 (the University of Minnesota, Minneapolis, MN, USA).^41,42^ The Nutrition Data Systems for Research provides nutrient information for >140 nutrients and compounds, including energy, saturated fat, and sodium. We measured diet quality with the HEI-2010, which was created by the US Department of Agriculture (Washington DC) and the National Cancer Institute and aligns with the 2010 US Dietary Guidelines for Americans.^39^ We calculated HEI-2010 scores using diet data in terms of Food Patterns Equivalents Database (FPED) by using a customized link^14^ between NDSR and FPED. FPED equivalents translate foods, as eaten, into standardized quantities of dietary components of interest; for example, an equivalent is an amount considered nutritionally equal to 1 cup in the vegetable, fruit, and dairy components or 1 ounce (1 ounce=28.35 g) in the grains or protein foods components.

The HEI-2010 is calculated by summing 12 component scores, each of which reflects recommendations in the 2010 Dietary Guidelines for Americans.^30^ Component scores are calculated using a density-based approach (i.e., per 1,000 kcal or percent of total energy), and higher scores for all components reflect closer adherence to the Dietary Guidelines. One component, empty calories (calories from solid fats and added sugars that add no nutrients) is scored from 0 to 20. Five components (whole grains, dairy, fatty acids, refined grains, and sodium) are scored from 0 to 10, and six components (total fruit, whole fruit, total vegetables, greens and beans, total protein foods, and seafood and plant proteins) are scored from 0 to 5. Components to be consumed in moderation—refined grains, sodium, and empty calories—are reverse scored. Thus the possible range for the total HEI score (the sum of the 12 components) is 0–100.^30^ For each participant in the LEAN study, the 12 HEI component scores and a total HEI score were calculated for both the baseline and 6-month dietary assessments. In addition to HEI scores, diet components that were specifically emphasized in the LEAN intervention were also used to evaluate participants’ diet quality. These diet components were derived from the FFQ and included calories, percent energy from fat, percent energy from saturated fat, added sugar, fiber, fruit servings, and vegetable servings.

### Statistical analysis

Due to the similarity of intervention components and weight loss outcomes between the in-person and telephone-based counseling groups,^25^ these two groups were combined for the purposes of these analyses. Characteristics of the usual care group and intervention groups were compared using *t*-tests for continuous variables and *Χ*^2^-tests or Fisher's exacts tests for categorical variables.

For the HEI score and all dietary intake variables, the main outcome was changed over the intervention period, which was calculated as the 6-month value minus the baseline value. To test for differences in average change between the two groups, generalized linear models were used with adjustment for baseline values. To assess whether a change in HEI score was associated with weight loss, participants within each group were categorized by their percentage of weight lost (<5% or ⩾5%) over the course of the intervention period. Analysis of variance was used to compare the change in HEI score by weight loss percent within each group, with adjustment for baseline HEI score.

## Figures and Tables

**Table 1:** Baseline participant characteristics

	*Usual care (*n*=30)* n *(%)*	*Intervention (*n*=51)* n *(%)*	P* value*^a^
Age (mean±s.d.)	57.90±7.41	59.24±7.15	0.426
BMI (mean±s.d.)	33.99±7.84	32.78±6.40	0.453
White	27 (90.0)	47 (94.0)	0.667
Latino	0 (0.0)	2 (3.9)	0.528
College graduate	20 (66.7)	35 (68.6)	0.855
Currently married	17 (56.7)	34 (66.7)	0.368
Postmenopausal	23 (76.7)	42 (82.4)	0.535
Ever smoker	13 (43.3)	20 (39.2)	0.716
Drink alcohol	23 (76.7)	48 (96.0)	0.008
1st degree family history of breast cancer	9 (30.0)	16 (32.0)	0.852
*Stage*			0.946
0	6 (20.0)	8 (15.7)	
I	15 (50.0)	26 (51.0)	
II	6 (20.0)	13 (25.5)	
III	2 (6.7)	3 (5.9)	
Unknown	1 (3.3)	1 (2.0)	
*Treatment*			0.868
Neither chemo nor radiation	5 (16.7)	7 (13.7)	
Radiation only	12 (40.0)	17 (33.3)	
Chemo only	4 (13.3)	9 (17.7)	
Both radiation and chemo	9 (30.0)	18 (35.3)	
Current endocrine therapy	24 (82.8)	40 (83.3)	0.948

a *T*-tests for continuous variables and *χ*^2^-test for categorical variables.

**Table 2:** Comparison of dietary components and HEI-2010 scores between intervention (*n*=51) and usual care (*n*=30)

	*Baseline (mean±s.d.)*	*6 months (mean±s.d.)*	*Change (mean±s.d.)*	P* value*^a^
*Calories*				0.561
Intervention	1,836.03±784.92	1,481.07±466.74	−354.96±725.65	
Usual care	1,762.25±747.39	1,522.64±674.85	−239.61±680.25	
*% Energy from fat*				0.013
Intervention	32.22±8.26	28.03±6.26	−4.19±6.49	
Usual care	31.99±6.92	30.87±5.76	−1.12±6.72	
*Fat (g)*				0.096
Intervention	67.84±37.40	46.43±19.84	−21.41±34.37	
Usual care	64.10±32.21	52.99±25.09	−11.12±27.93	
*% energy from saturated fat*				0.003
Intervention	10.29±2.91	8.09±2.24	−2.20±2.39	
Usual care	10.34±2.73	9.29±1.79	−1.05±2.16	
*Added sugar (gm per 1,000 kcal)*				0.084
Intervention	30.04±15.38	25.14±10.18	−4.90±14.95	
Usual care	32.76±10.72	30.33±13.47	−2.44±9.37	
*Fiber (gm per 1,000 kcal)*				0.007
Intervention	12.22±3.84	17.06±4.98	4.84±4.72	
Usual care	12.56±4.29	13.83±4.27	1.28±4.21	
*Fruit servings*				0.006
Intervention	2.18±1.43	2.84±1.81	0.66±1.66	
Usual care	2.35±1.77	1.98±1.33	−0.37±1.51	
*Vegetable servings*				0.106
Intervention	2.79±1.54	3.32±1.74	0.53±1.58	
Usual care	2.72±1.79	2.74±2.02	0.02±1.20	
*HEI-2010 score*				0.092
Intervention	70.17±8.51	76.97±9.80	6.80±10.06	
Usual care	71.08±9.27	74.13±9.09	3.05±8.03	

Abbreviation: HEI, healthy eating index.

a *P* value for difference between groups in change in intake (6 months—baseline), controlling for baseline.

**Table 3:** Change in HEI score by percentage of weight loss

	*Weight loss <5%*	*Weight loss ⩾5%*	P* value*^a^
Intervention^b^	2.82±7.20	10.35±11.09	0.010
Usual care^c^	2.38±8.15	4.89±7.90	0.268

Abbreviation: HEI, healthy eating index.

a *P* value for difference in change in HEI score by weight loss %, within each group, controlling for baseline HEI score.

b Intervention group: weight loss <5% (*n*=23); weight loss ⩾5% (*n*=27).

c Usual care group: weight loss <5% (*n*=22); weight loss ⩾5% (*n*=8).
